# Evaluation of Oral Subchronic Toxicity of Soshiho-Tang Water Extract: The Traditional Herbal Formula in Rats

**DOI:** 10.1155/2013/590181

**Published:** 2013-03-20

**Authors:** Mee-Young Lee, Chang-Seob Seo, In-Shik Shin, Young-Bum Kim, Jung-Hoon Kim, Hyeun-Kyoo Shin

**Affiliations:** ^1^Basic Herbal Research Group, Korea Institute of Oriental Medicine, Exporo 483, Yuseong-gu, Daejeon 305-811, Republic of Korea; ^2^Division of Nonclinical Studies, Korea Institute of Toxicology, P.O. Box 123, 19 Sinseongro, Yuseong-gu, Daejeon 305-343, Republic of Korea

## Abstract

Soshiho-tang (Xiao-chai-hu-tang in Chinese and Sho-saiko-to in Japanese) has been widely used for its various pharmacological effects, which include anti-inflammatory, antioxidant, antihepatic fibrosis, and antitumor properties. To evaluate the safety of Soshiho-tang water extract (SST), we tested its subchronic toxicity in male and female Crl:CD (SD) rats. Rats were orally treated with four different doses (0, 500, 1000, and 2000 mg/kg/day) of SST administered for 13 weeks. Mortality, clinical signs, body weight changes, food and water consumption changes, ophthalmology, urinalysis, hematological and biochemical parameters, gross findings, organ weights, and histological markers were monitored during the study. The SST treatment did not result in any toxicologically significant changes in mortality, clinical signs, body weights, food and water consumption, ophthalmoscopy, urinalysis, hematological and serum biochemical parameters, gross findings, organ weights, or histopathology. Histological analysis did not show any liver or kidney alteration. We concluded that the 13-week repeated oral administration of SST did not cause any adverse effects in rats at dosage levels of ≤2000 mg/kg/day. Under these experimental conditions, the no-observed-adverse-effect level was concluded to be 2000 mg/kg/day for both sexes.

## 1. Introduction

Medicinal herbs are used for the prevention and treatment of disease and have a long history. Because traditional herbal medicines have few side effects and are very effective, the consumption of these medicines has increased substantially in recent decades [1]. With increasing herbal medicine consumption, concerns have been raised over the lack of quality control and scientific evidence of the efficacy and safety of herbal medicine [2–4].

Soshiho-tang (Xiao-chai-hu-tang in Chinese and Sho-saiko-to in Japanese) is frequently used for the treatment of liver disorders. It is composed of seven herbs: Bupleuri Radix, Scutellariae Radix, Ginseng Radix, Pinelliae Tuber, Glycyrrhizae Radix et Rhizoma, Zingiberis Rhizoma Crudus, and Zizyphi Fructus (Table 1). According to previous studies, Soshiho-tang exhibits various pharmacological properties including anti-inflammatory [5], antioxidant [6], immunomodulating [7], hepatoprotective [8], antihepatic fibrosis [9], and antitumor effects [10]. Although Soshiho-tang has various pharmacological properties, few scientific studies have investigated its safety and toxicity. Recently, the complexity of herbal prescriptions in general, and their inherent biological variations, was demonstrated; it is now necessary to evaluate their safety, efficacy, and quality [1]. However, the toxicity and quality control of herbal formulas to the public is becoming a problem because of the lack of scientific evidence about herbal formulas. Because few studies have explored the safety and toxicity of herbal formulas, public concern has been raised regarding their potential adverse effects [11].

The present study examined the safety of the oral use of SST for 13 weeks. The study was conducted in accordance with the guidelines established by the Organization for Economic Cooperation and Development (OECD, TG 408) [12] for the testing of chemicals and recent Good Laboratory Practice (GLP) Regulations.

## 2. Materials and Methods

### 2.1. Reagents and Materials

Liquiritin, baicalin, and glycyrrhizin were purchased from Wako Pure Chemical Industries, Ltd. (Osaka, Japan). The purity of each component was determined to be above 98% by high-performance liquid chromatography (HPLC) analysis. HPLC-grade reagents, methanol, acetonitrile, and water were obtained from J. T. Baker (Phillipsburg, NJ, USA). Glacial acetic acid was of analytical reagent grade, procured from Junsei (Tokyo, Japan). The materials used to form Soshiho-tang were purchased from Omniherb (Yeongcheon, Republic of Korea) and HMAX (Chungbuk, Republic of Korea). A voucher specimen (2008-KE26-1~KE26-7) was deposited at the Basic Herbal Medicine Research Group, Korea Institute of Oriental Medicine.

### 2.2. Preparation of Soshiho-Tang Water Extract (SST) and Sample Solutions

A decoction of Soshiho-tang was prepared in our laboratory from a mixture of chopped crude herbs (Table 1), extracted in distilled water at 100°C for 2 h. The solution was evaporated to dryness, and freeze-dried (yield: 22.9%). Lyophilized Soshiho-tang extract (200 mg) was dissolved in distilled water (20 mL). The solution was then diluted tenfold with distilled water. The solution was filtered using a SmartPor GHP syringe filter (0.2 *μ*m, Woongki Science, Seoul, Republic of Korea).

### 2.3. Preparation of Standard Solutions

A methanol standard stock solution containing liquiritin, baicalin, and glycyrrhizin (all 1.0 mg/mL) was prepared and diluted to the appropriate concentration range for the establishment of calibration curves. The calibration curves were calculated using the peak areas of standard solution in the range 3.91–250.00 *μ*g/mL for the three compounds.

### 2.4. HPLC Analysis of SST

A Shimadzu LC-20A HPLC system (Shimadzu Co., Kyoto, Japan) consisting of a solvent delivery unit, an online degasser, a column oven, an autosampler, and a PDA detector was used. The data processor employed LCsolution software (version 1.24). The analytical column used was a Gemini C18 (250 × 4.6 mm; particle size 5 *μ*m, Phenomenex, Torrance, CA, USA). The mobile phases consisted of solvent A (1.0%, v/v, aqueous acetic acid) and solvent B (acetonitrile with 1.0%, v/v, acetic acid). The gradient flow was as follows: (A)/(B) = 85/15 (0 min) → (A)/(B) = 60/40 (20 min) → (A)/(B) = 45/55 (40 min) → (A)/(B) = 0/100 (50 min; hold for 5 min) → (A)/(B) = 85/15 (55 min; hold for 15 min). The column temperature was maintained at 30°C. The analysis was carried out at a flow rate of 1.0 mL/min with PDA detection at 254 and 275 nm. The injection volume was 10 *μ*L.

### 2.5. Experimental Animals and Design

Forty-eight Crl:CD (SD) rats of each sex were obtained from a specific pathogen-free facility at Orient Bio Co. (Seoul, Republic of Korea) at 5 weeks of age and used after a week of quarantine and acclimatization. This study was approved by Korea Institute of Oriental Medicine Institutional Animal Care and Use Committee and was performed at the Korea Institute of Toxicology (Daejeon, Republic of Korea) and conducted according to the guidance of the Institutional Animal Care and Use Committee in Korea Institute of Toxicology (KRICT) (accredited by AAALAC International, 1998) under the GLP Regulations for Nonclinical Laboratory Studies.

Healthy male and female rats were randomly assigned to four experimental groups: SST 0, 500, 1000, and 2000 mg/kg/day groups and a vehicle control group. Each experimental group consisted of 10 rats of each sex.

In the previous 4-week repeated-dose study of SST, no treatment-related toxic changes were observed at the highest dose of 2000 mg/kg/day. Therefore, the highest dose level of 2000 mg/kg/day, with a common ratio of 2, was chosen for this 13-week repeated-dose study. Oral administration was selected in the present study, because the oral route is the clinically intended route for the administration of Soshiho-tang. The SST was suspended in distilled water and was freshly prepared daily before treatment for 13 weeks. The daily application volume (10 mL/kg of body weight) of SST was calculated in advance, based on the most recently recorded body weights of individual animals.

### 2.6. Clinical Observations and Mortality

All animals were observed twice daily and clinical signs and mortality (if any) were recorded. Body weight and food and water intake were monitored weekly. After a one-week acclimation period, rats were observed twice daily (before and after the daily treatment) for the presence of common symptoms and mortality. Food and water consumption was recorded once before treatment onset and approximately once a week thereafter by measuring the difference between the amounts provided, and amounts remaining on the following day. This was regarded as daily consumption. External eye examinations were performed during the pretest period and all animals in each group were briefly examined via both external and fundus examination using an indirect binocular ophthalmoscope (IO-H, Neitz Instruments Co., Japan) during the last week of dosing. Mortality and clinical signs were recorded using the PATH/TOX SYSTEM (version 4.2.2).

### 2.7. Urinalysis, Hematology, and Serum Biochemistry

Fresh urine was collected from all animals, and urinalysis was conducted to assess urine volume, specific gravity, pH, and the levels of protein, ketone bodies, glucose, bilirubin, nitrite, urobilinogen, color, cast, epithelial cells, erythrocytes, leucocytes, and occult blood using a Multistix 10SG instrument (Bayer, USA) and a urine chemical analyzer (CliniTek 500, Bayer, USA).

Fresh blood samples were analyzed to determine the red blood cell count, hemoglobin concentration, hematocrit level, mean corpuscular cell volume, mean corpuscular cell hemoglobin, mean corpuscular cell hemoglobin concentration, platelet count, white blood cell count (WBC), differential WBC count, reticulocyte count, prothrombin time, and activated partial thromboplastin time. All parameters were measured using an ADVIA 120 Hematology System (Bayer, USA). In addition, prothrombin time and activated partial thromboplastin time were determined from blood samples treated with 3.2% sodium citrate, using a coagulometer (Coagrez-100s, Japan).

Serum biochemistry parameters were examined, including the levels of blood urea nitrogen (BUN), alanine aminotransferase (ALT), aspartate aminotransferase (AST), alkaline phosphatase (ALP), creatinine (CREA), glucose (GLU), total cholesterol (TCHO), albumin/globulin ratio (A/G), total protein (TP), albumin (ALB), creatine kinase (CK), triglycerides (TG), total bilirubin (TBIL), phospholipids (PL), calcium (Ca), inorganic phosphorus (IP), chloride (Cl), sodium (Na), and potassium (K).

### 2.8. Necropsy

Following blood collection, the organs (brain, pituitary gland, adrenal gland, liver, spleen, kidneys, heart, thymus, lungs, salivary glands, thyroid glands, testes, epididymides, seminal vesicles, prostate, uterus, and ovaries) were removed, weighed, and examined macroscopically. The absolute weight was measured and the relative organ weight (percentage of body weight) was calculated.

### 2.9. Histopathological Examination

The testes and epididymides were fixed with Bouin's solution. The following samples were fixed using a 10% formalin solution: kidneys, urinary bladder, liver, heart, spleen, thymus, salivary glands, aorta, pancreas, tongue, skeletal muscle, sciatic nerve, brain, thoracic SC, eyes and optic nerve, lungs, trachea, thyroid, parathyroid, adrenal gland, mandibular lymph node, the Harderian glands, esophagus, stomach, duodenum, jejunum, ileum, cecum, colon, rectum, mesenteric lymph node skin, mammary gland, sternum/marrow, femur/marrow, testes, epididymides, prostate, seminal vesicle, ovaries, uterus/cervix, and vagina. All samples were sectioned and stained with hematoxylin and eosin.

### 2.10. Statistical Analyses

Data collected during the study were examined for variance homogeneity using Bartlett's test. One-way ANOVA was performed at *α* = 0.05 in cases in which Bartlett's test indicated no significant deviations from variance homogeneity. When significance was noted, a multiple comparison test (Dunnett's test) was used to determine which pairs of groups were significantly different. In cases in which significant deviations from variance homogeneity were observed, a nonparametric comparison test (Kruskal-Walli's test) was performed. When a significant difference was observed in the Kruskal-Wallis test, Dunn's rank sum test was used to determine the specific pairs. Statistical analyses were performed using the PATH/TOX SYSTEM (version 4.2.2, Xybion Medical Systems Corporation, USA) and the SAS program (version 9.2). The level of significance was set at *P* < 0.05 or 0.01. Because treatment-related animal deaths were not observed, LD_50_ values were not measured.

## 3. Results

### 3.1. Linearity, Range, Limit of Detection (LOD), and Limit of Quantification (LOQ)

Calibration curves of liquiritin, baicalin, and glycyrrhizin were obtained using standard solutions. The coefficients (*R* ^2^) of the calibration curves for the three constituents were ≥0.9993 (Table 2). LOD and LOQ were determined based on signal-to-noise (S/N) ratios of 3 and 10, respectively. The LOD and LOQ values were 0.09 and 0.29 *μ*g/mL for liquiritin, 0.41 and 1.37 *μ*g/mL for baicalin, and 0.21 and 0.70 *μ*g/mL for glycyrrhizin.

### 3.2. HPLC Analysis of SST

**Figure 1:**
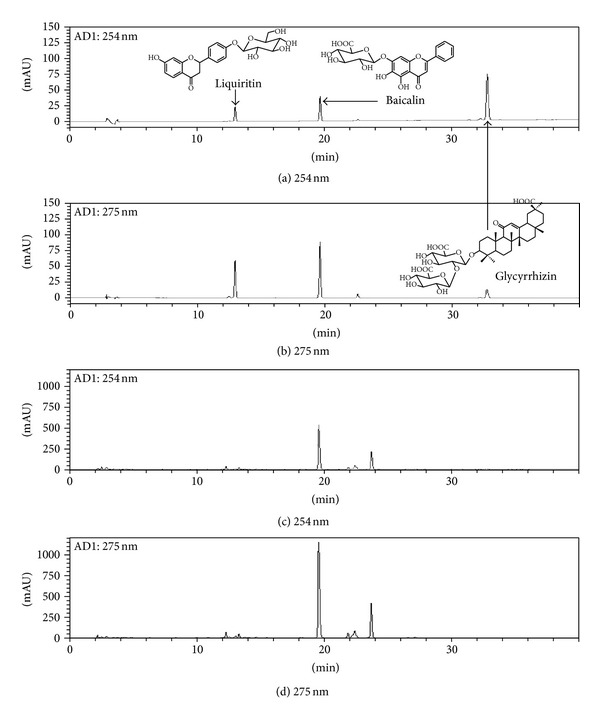
HPLC chromatogram of the standard mixtures ((a) and (b)) and SST samples ((c) and (d)).

Our analysis method was applied to the simultaneous determination of three compounds in SST: liquiritin, baicalin, and glycyrrhizin. The retention times of the three compounds were 12.99, 19.64, and 32.71 min for liquiritin, baicalin, and glycyrrhizin, respectively. The stability test was analyzed using the sample solution at 0, 1, 2, 5, 7, and 10 days. The relative standard deviation values of retention time and peak area were within 1.12% and 3.09%, respectively (Table 2). These results suggest that the solution was stable for at least 10 days.  Figure 1 shows chromatograms of reference components and SST, with detection of eluents at 254 and 275 nm. The concentrations of the three components identified in SST were 2.33–61.58 mg/g. These results are summarized in Table 3.

### 3.3. Mortality and Clinical Signs

There was no mortality attributed to any effect of SST during the 13 weeks of drug administration. No SST treatment-related clinical signs were detected, with the exception of loss of fur, bite wounds, scratch wounds, eye discharge, and scabs.

### 3.4. Body Weight and Food Consumption

**Figure 2:**
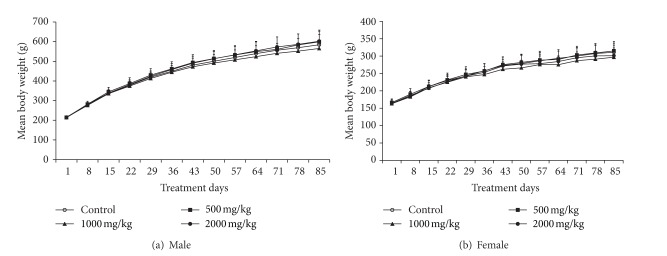
Mean body weight changes in male (a) and female (b) rats treated with SST at dose levels of 0 (∘), 500 (■), 1000 (▲), and 2000 (●) mg/kg/day for 13 weeks. Data are presented as mean values ± SD.

There were no significant differences in body weight (Figures 2(a) and 2(b)) or food consumption between control and treatment groups. Also, there was no clear food consumption in SST-treated groups compared with control group (data not shown).

### 3.5. Ophthalmological Examination and Urinalysis

There were no abnormal ophthalmologic findings among the SST-treated groups compared with each sex of the control group in the present study (data not shown). There were no significant changes in urinalysis results in any of the SST-treated groups compared with each sex of the control group (Table 4).

### 3.6. Hematology

The results of the hematological examination are shown in Table 5. No treatment-related changes in hematological parameters were observed in the present study. A prothrombin time delay was observed in the 2000 mg/kg/day male groups.

### 3.7. Serum Biochemistry

The results of the examination of serum biochemical values in the SST-treated and control groups are shown in Table 6. In the male 1000 mg/kg/day group, the levels of TBIL were significantly decreased. No other toxic-related serum biochemical value changes were observed in any group.

### 3.8. Relative Organ Weight

The results of relative organ weights are shown in Table 7. There were no significant differences related to SST treatment in the relative organ weight of male or female rats. In females, the relative kidney weight was significantly increased in the 1000 and 2000 mg/kg/day groups, and the relative liver and heart weights were significantly increased in the 2000 mg/kg/day group.

### 3.9. Histopathology

No treatment-related histopathology findings were observed after 13 weeks of SST treatment, and there were no histologic correlates for the organ weight changes (data not shown). Histopathological changes included minimal cardiomyopathy of the heart and foamy macrophages of the lungs in the male 2000 mg/kg/day group, and genital organs and estrus cycles were increased in the female group compared with the control group. These changes were not considered to be treatment related, because these microscopic changes are commonly observed in untreated laboratory animals and the incidence and severity were comparable between the treated and control animals.

**Figure 3:**
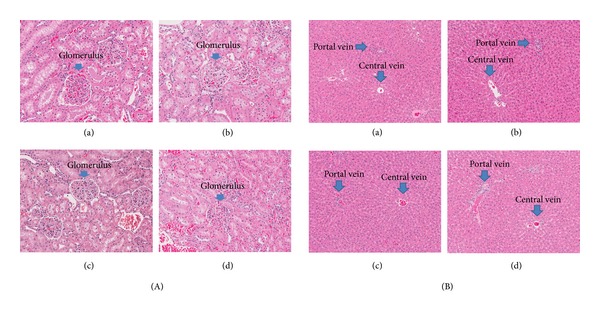
Histopathological findings in the kidney (A) and liver (B). (A) (a) Control male group, (b) 2000 mg/kg/day SST-treated male group, (c) control female group, and (d) 2000 mg/kg/day SST-treated female group. H&E stain; magnification, 200x.

Histopathological features of the kidney (Figure 3(A)) and liver (Figure 3(B)) in the control group and the highest dosage SST-treated groups showed normal structure.

## 4. Discussion

Herbal medicines have become increasingly popular in modern societies around the globe, and many research projects have conducted the studies on herbal medicine's pharmacological properties to establish its scientific evidence. Evidence of therapeutic materials is assessed throughout various sources including toxicity, pharmacological properties, clinical trials, and systematic reviews [13]. Traditional herbal medicine is the most important part of complementary and alternative medicine and has been practiced for thousands of years. Although there are many traditional herbal medicines available, and some have been verified by clinical trials, their efficacy and safety are still questioned by consumers [14].

Some traditional herbal preparations in Korea and Japan are officially recognized as approved ethical drugs, and, in some cases, are covered by the National Health Insurance Program. Therefore, to confirm the safety of SST, the present study assessed its repeated-dose oral toxicity for 13 weeks in female and male Crl:CD (SD) rats. The mortality and changes in body weight, food consumption, clinical signs, urinalysis, ophthalmological examination, hematology, serum biochemistry, gross observation, organ weight, and histopathology were monitored in accordance with the KFDA and OECD guidelines. Overall, SST was not associated with adverse effects in doses up to 2000 mg/kg/day administered for up to 13 weeks in both sexes.

Herbal medicine, a mixture of several herbs are considered to decrease its toxicity and enhance or prolong the pharmacological effects of any component. Toxicity assessment of SST has been studied in Japan. Previous study demonstrated that administration of SST did not cause any adverse effects at 1000 mg/kg/day [15]. However, dose of SST in previous study was comparatively lower than that used in our study, and toxicity study has been conducted under non-GLP conditions. Therefore, the present study has been conducted to clarify the safety of SST at GLP Regulations. 

Clinical signs of salivation were observed in female (*n* = 10) and male (*n* = 2) 2000 mg/kg/day SST-treated rats. However, these were not considered to be related to SST treatment because incidence was low and no dose-response relationship was observed.

There were no changes in body weight, food consumption, urinalysis, or ophthalmology. No abnormal changes were detected regarding hematological and serum biochemical parameters up to the highest dosage of SST, 2000 mg/kg/day; the changes in both sexes of treated rats were comparable to the changes in both sexes of control group rats.

Statistically significant increases in relative liver, kidney, and heart weights were observed in female rats treated with SST. However, these changes were not correlated with the SST dosage and were within the normal range. Moreover, no gross pathological findings or histopathological changes were observed. Therefore, these changes in body weight were not considered to be SST-induced abnormalities.

Histopathological findings showed cardiomyopathy of the heart and foamy macrophages in the lungs in the male 2000 mg/kg/day rats. In the female 2000 mg/kg/day rats there was an increase in the estrus cycle compared with the control group. However, there were no histopathological findings for the ovaries or uterus. Although histopathological lesions were sporadically observed in the organs in male and female SST-treated rats, the degree and incidence were similar to those of the control groups. Moreover, these findings are commonly observed in normal rats [16–18]. Therefore, it is reasonable to conclude that they were of little toxicological significance.

In conclusion, repeated oral dose administration of SST to SD rats for 13 weeks did not cause any adverse effects in rats of either sex, up to and including doses of 2000 mg/kg/day. Thus, the no-observed-adverse-effect level was concluded to be 2000 mg/kg/day for male and female rats.

## Figures and Tables

**Table 1:** Composition of Soshiho-tang.

Latin name	Amount (g)	Company of purchase	Source
Bupleuri Radix	11.25	HMAX	China
Scutellariae Radix	7.5	HMAX	Jeongseon, Korea
Pinelliae Tuber	3.75	HMAX	China
Ginseng Radix Alba	3.75	Omniherb	Geumsan, Korea
Zizyphi Fructus	3.75	Omniherb	Yeongcheon, Korea
Zingiberis Rhizoma Crudus	3.75	Omniherb	Yeongcheon, Korea
Glycyrrhizae Radix et Rhizoma	1.875	HMAX	China

Total	35.625		

**Table 2:** Regression data, linear range, correlation coefficient, and stability for three compounds (*n* = 3).

Compound	Linear range (*μ*g/mL)	Slope	Intercept	Correlation coefficient (*R* ^2^)	Stability (RSD (%))	
					Retention time	Peak area
Liquiritin	3.91–250.00	20031	−19996	0.9997	0.21	3.09
Baicalin	3.91–250.00	38806	−79724	0.9993	1.12	0.82
Glycyrrhizin	3.91–250.00	8959.4	−15143	0.9996	1.10	1.05

**Table 3:** Contents of three components in the SST for 13 weeks by HPLC (*n* = 3).

Compound	0 week			1 week			13 week		
	Mean (mg/g)	SD	RSD (%)	Mean (mg/g)	SD	RSD (%)	Mean (mg/g)	SD	RSD (%)
Liquiritin	2.71	0.011	0.418	2.60	0.014	0.534	2.56	0.010	0.399
Baiclin	61.58	0.022	0.036	58.02	0.197	0.339	58.01	0.061	0.105
Glycyrrhizin	2.33	0.003	0.113	2.44	0.012	0.486	2.45	0.021	0.856

**Table 4:** Urinalysis values with SST for 13 weeks.

Parameters	Male				Female			
	Control	500 mg/kg	1000 mg/kg	2000 mg/kg	Control	500 mg/kg	1000 mg/kg	2000 mg/kg
Volume (mL)	18 ± 6.8	17 ± 7.0	15 ± 7.3	19 ± 5.2	9 ± 4.2	11 ± 7.3	10 ± 9.1	8 ± 3.4
SG	1.08 ± 0.0059	1.02 ± 0.0058	1.02 ± 0.0005	1.01 ± 0.0041	1.02 ± 0.0058	1.01 ± 0.0035	1.02 ± 0.0081	1.02 ± 0.0071
pH	7.0 ± 0.28	7.0 ± 0.16	6.8 ± 0.26	7.0 ± 0.16	6.7 ± 0.26	6.9 ± 0.21	6.5 ± 0.41	6.8 ± 0.26
URO (E.U./dL)	0.2 ± 0.00	0.2 ± 0.00	0.2 ± 0.00	0.2 ± 0.00	0.2 ± 0.00	0.2 ± 0.00	0.2 ± 0.00	0.2 ± 0.00

**Table 5:** Hematological values of rats treated orally with SST for 13 weeks.

Parameters	Male				Female			
	Control	500 mg/kg	1000 mg/kg	2000 mg/kg	Control	500 mg/kg	1000 mg/kg	2000 mg/kg
WBC (×10^3^/*μ*L)	13.50 ± 3.755	11.19 ± 3.405	10.88 ± 2.983	11.46 ± 2.415	8.30 ± 2.100	8.61 ± 2.266	7.93 ± 2.220	8.84 ± 2.155
RBC (×10^6^/*μ*L)	9.13 ± 0.353	9.14 ± 0.445	9.38 ± 0.349	9.00 ± 0.265	8.59 ± 0.312	8.43 ± 0.437	8.44 ± 0.383	8.50 ± 0.354
HGB (g/dL)	16.2 ± 0.77	16.4 ± 0.57	16.6 ± 0.64	16.1 ± 0.44	16.0 ± 0.38	16.1 ± 0.57	16.2 ± 0.77	16.3 ± 0.74
HCT (%)	49.1 ± 2.00	50.1 ± 1.62	50.1 ± 2.11	48.6 ± 1.17	48.0 ± 1.55	47.7 ± 1.99	47.5 ± 2.12	48.0 ± 1.91
MCV (fL)	53.8 ± 1.72	54.9 ± 1.98	53.4 ± 1.45	54.1 ± 1.56	55.9 ± 1.31	56.6 ± 1.12	56.3 ± 2.37	56.5 ± 1.91
MCH (pg)	17.7 ± 0.91	18.0 ± 0.84	17.7 ± 0.50	17.9 ± 0.72	18.7 ± 0.53	19.1 ± 0.54	19.2 ± 0.88	19.2 ± 0.74
MCHC (g/dL)	32.9 ± 0.98	32.8 ± 0.56	33.1 ± 0.37	33.1 ± 0.61	33.4 ± 0.75	33.8 ± 0.74	34.1 ± 0.45	34.0 ± 0.84
PLT (×10^3^/*μ*L)	1110 ± 84.7	1061 ± 124.4	1038 ± 122.2	1086 ± 94.6	1082 ± 62.3	1072 ± 142.6	1025 ± 108.8	1196 ± 550.6
Reticulocyte (%)	2.5 ± 0.41	2.3 ± 0.33	2.3 ± 0.43	2.3 ± 0.39	2.5 ± 0.42	2.5 ± 0.29	2.1 ± 0.47	2.8 ± 0.98
Neutrophils (%)	15.09 ± 5.508	16.58 ± 5.953	19.51 ± 7.614	17.27 ± 5.569	12.50 ± 4.448	13.24 ± 4.517	11.28 ± 4.780	12.42 ± 4.247
Lymphocytes (%)	79.2 ± 5.78	77.7 ± 5.60	74.8 ± 8.29	76.5 ± 6.17	81.8 ± 4.52	80.7 ± 5.28	82.5 ± 4.97	82.4 ± 4.55
Monocytes (%)	3.2 ± 0.98	3.1 ± 0.66	2.9 ± 1.12	3.3 ± 0.74	2.8 ± 0.69	3.2 ± 0.85	3.2 ± 0.58	2.6 ± 0.64
Eosinophils (%)	1.1 ± 0.28	1.2 ± 0.40	1.4 ± 0.44	1.2 ± 0.29	1.2 ± 0.25	1.3 ± 0.60	1.3 ± 0.32	1.1 ± 0.33
Basophils (%)	0.6 ± 0.22	0.8 ± 0.22	0.7 ± 0.21	0.8 ± 0.23	0.7 ± 0.30	0.6 ± 0.28	0.7 ± 0.28	0.6 ± 0.26
LUC (%)	0.9 ± 0.31	0.8 ± 0.24	0.8 ± 0.22	1.1 ± 0.56	1.0 ± 0.22	1.0 ± 0.27	1.0 ± 0.22	0.8 ± 0.22
PT (sec)	15.9 ± 1.05	16.2 ± 0.71	16.8 ± 1.20	17.2 ± 1.13*	15.5 ± 0.78	15.2 ± 0.53	16.1 ± 1.77	16.2 ± 1.21
APTT (sec)	16.8 ± 1.52	17.1 ± 0.79	17.3 ± 0.82	17.1 ± 0.87	14.6 ± 1.22	14.4 ± 0.99	14.5 ± 1.32	14.3 ± 1.50

*Significant differences from control group (*P* < 0.05).

**Table 6:** Serum biochemical values of rats treated orally with SST for 13 weeks.

Parameters	Male				Female			
	Control	500 mg/kg	1000 mg/kg	2000 mg/kg	Control	500 mg/kg	1000 mg/kg	2000 mg/kg
GLU (mg/dL)	137.4 ± 44.22	121.6 ± 24.25	111.5 ± 20.60	125.2 ± 35.90	155.3 ± 27.24	138.2 ± 18.97	128.8 ± 29.05	144.5 ± 21.23
BUN (mg/dL)	14.6 ± 1.45	14.4 ± 1.31	13.8 ± 1.92	14.7 ± 1.02	18.7 ± 2.38	16.0 ± 1.95	17.5 ± 2.19	17.1 ± 2.32
CREA (mg/dL)	0.56 ± 0.052	0.55 ± 0.061	0.57 ± 0.063	0.56 ± 0.052	0.71 ± 0.046	0.69 ± 0.050	0.69 ± 0.054	0.69 ± 0.086
TP (g/dL)	6.98 ± 0.387	6.96 ± 0.228	6.85 ± 0.290	7.08 ± 0.283	7.86 ± 0.374	7.93 ± 0.459	7.78 ± 0.470	8.13 ± 0.584
ALB (g/dL)	4.49 ± 0.156	4.49 ± 0.108	4.40 ± 0.116	4.54 ± 0.166	5.21 ± 0.265	5.28 ± 0.258	5.23 ± 0.262	5.38 ± 0.310
A/G (ratio)	1.82 ± 0.149	1.83 ± 0.125	1.81 ± 0.151	1.80 ± 0.148	1.97 ± 0.093	2.00 ± 0.112	2.06 ± 0.086	1.97 ± 0.136
TCHO (mg/dL)	70.3 ± 22.49	71.8 ± 12.37	64.9 ± 14.67	73.8 ± 16.34	91.4 ± 24.91	89.6 ± 17.10	95.2 ± 18.61	91.3 ± 24.44
TG (mg/dL)	55.4 ± 22.90	55.1 ± 16.17	50.6 ± 14.86	67.2 ± 35.73	60.7 ± 27.67	42.1 ± 8.77	47.3 ± 10.10	51.2 ± 14.93
PL (mg/dL)	102 ± 22.7	107 ± 16.5	96 ± 15.7	110 ± 22.8	169 ± 38.3	162 ± 20.9	170 ± 25.9	169 ± 36.2
AST (IU/L)	124.9 ± 29.29	117.1 ± 13.76	122.3 ± 18.48	114.1 ± 14.50	116.6 ± 34.14	142.1 ± 73.28	138.1 ± 29.01	115.1 ± 22.97
ALT (IU/L)	35.9 ± 8.43	33.6 ± 5.45	33.1 ± 8.90	33.6 ± 5.25	32.5 ± 10.83	49.3 ± 46.80	49.5 ± 14.82	35.6 ± 14.54
TBIL (mg/dL)	0.157 ± 0.01	0.141 ± 0.01	0.133 ± 0.02*	0.149 ± 0.01	0.178 ± 0.01	0.183 ± 0.02	0.154 ± 0.02	0.192 ± 0.03
ALP (IU/L)	214.1 ± 29.52	241.9 ± 28.77	241.2 ± 40.68	230.2 ± 25.28	124.2 ± 34.46	117.5 ± 25.35	137.5 ± 46.11	125.0 ± 21.91
CK (IU/L)	623 ± 197.7	579 ± 154.9	622 ± 162.4	581 ± 212.6	550 ± 244.5	549 ± 138.0	534 ± 209.3	468 ± 162.6
Ca (mg/dL)	11.08 ± 0.36	11.22 ± 0.37	11.03 ± 0.38	11.49 ± 0.45	11.88 ± 0.51	11.83 ± 0.36	11.71 ± 0.46	12.30 ± 0.49
IP (mg/dL)	9.12 ± 1.48	9.13 ± 0.60	8.55 ± 0.65	9.29 ± 0.65	7.01 ± 1.069	7.13 ± 1.190	7.01 ± 1.185	7.95 ± 1.101
Na (mmol/L)	145 ± 2.7	146 ± 2.7	147 ± 2.3	148 ± 0.9	147 ± 2.3	146 ± 1.1	146 ± 1.8	147 ± 1.5
K (mmol/L)	8.26 ± 2.20	8.32 ± 1.49	7.43 ± 1.27	7.83 ± 0.69	6.81 ± 0.765	7.04 ± 1.075	7.22 ± 1.294	7.78 ± 1.469
CI (mmol/L)	101 ± 0.7	102 ± 1.5	102 ± 1.5	102 ± 1.6	104 ± 1.4	103 ± 1.3	104 ± 1.0	102 ± 1.4

*Significant differences from control group (*P* < 0.05).

**Table 7:** Relative organ weights of rats treated orally with SST (percentage of body weight) for 13 weeks.

Parameters	Male				Female			
	Control	500 mg/kg	1000 mg/kg	2000 mg/kg	Control	500 mg/kg	1000 mg/kg	2000 mg/kg
Brain	0.379 ± 0.03	0.372 ± 0.02	0.388 ± 0.02	0.378 ± 0.02	0.665 ± 0.049	0.644 ± 0.054	0.681 ± 0.056	0.681 ± 0.083
Pituitary gland	0.002 ± 0.0002	0.002 ± 0.0002	0.002 ± 0.0002	0.002 ± 0.0003	0.006 ± 0.0006	0.006 ± 0.0007	0.006 ± 0.0010	0.006 ± 0.0008
Liver	2.771 ± 0.216	2.718 ± 0.239	2.700 ± 0.224	2.848 ± 0.228	2.755 ± 0.165	2.784 ± 0.100	2.778 ± 0.112	2.938 ± 0.120^#^
Spleen	0.160 ± 0.023	0.157 ± 0.022	0.157 ± 0.018	0.163 ± 0.019	0.184 ± 0.025	0.185 ± 0.014	0.180 ± 0.024	0.198 ± 0.028
Heart	0.285 ± 0.026	0.276 ± 0.014	0.273 ± 0.019	0.269 ± 0.017	0.323 ± 0.021	0.315 ± 0.020	0.338 ± 0.019	0.347 ± 0.022*
Thymus	0.084 ± 0.022	0.072 ± 0.008	0.074 ± 0.009	0.140 ± 0.022	0.114 ± 0.035	0.109 ± 0.025	0.119 ± 0.012	0.120 ± 0.019
Salivary glands	0.137 ± 0.019	0.141 ± 0.018	0.140 ± 0.022	0.141 ± 0.013	0.167 ± 0.019	0.162 ± 0.013	0.172 ± 0.012	0.171 ± 0.022
Seminal vesicle	0.373 ± 0.054	0.388 ± 0.057	0.408 ± 0.049	0.347 ± 0.065				
Prostate	0.124 ± 0.039	0.128 ± 0.027	0.114 ± 0.027	0.118 ± 0.029				
Kidneys	0.695 ± 0.066	0.689 ± 0.073	0.696 ± 0.056	0.673 ± 0.043	0.699 ± 0.059	0.736 ± 0.037	0.758 ± 0.046*	0.774 ± 0.056^#^
Adrenal glands	0.013 ± 0.001	0.012 ± 0.001	0.011 ± 0.001	0.012 ± 0.001	0.028 ± 0.003	0.026 ± 0.002	0.027 ± 0.003	0.028 ± 0.004
Testis (female: ovary)	0.652 ± 0.07	0.619 ± 0.08	0.660 ± 0.05	0.644 ± 0.048	0.033 ± 0.006	0.034 ± 0.003	0.037 ± 0.005	0.038 ± 0.007
Epididymides (female: uterus/cervix)	0.281 ± 0.0318	0.276 ± 0.022	0.284 ± 0.018	0.290 ± 0.029	0.248 ± 0.061	0.208 ± 0.051	0.228 ± 0.052	0.238 ± 0.047
Lung	0.305 ± 0.027	0.302 ± 0.023	0.316 ± 0.014	0.296 ± 0.027	0.446 ± 0.04	0.427 ± 0.03	0.458 ± 0.03	0.507 ± 0.09
Thyroid/parathyroid	0.005 ± 0.001	0.005 ± 0.0009	0.005 ± 0.0013	0.005 ± 0.0011	0.007 ± 0.001	0.007 ± 0.001	0.006 ± 0.001	0.007 ± 0.001

*Significant differences from control group (*P* < 0.05) ^#^Significant differences from control group (*P* < 0.05).
